# Zhishi Xiebai Guizhi Decoction modulates hypoxia and lipid toxicity to alleviate pulmonary vascular remodeling of pulmonary hypertension in rats

**DOI:** 10.1186/s13020-024-01039-0

**Published:** 2024-12-19

**Authors:** Min Fu, Yuan Li, Jingjing Liu, Junjie Liu, Jiaoxia Wei, Yuxin Qiao, Hanxin Zhong, Dongyang Han, Haitao Lu, Li Yao

**Affiliations:** 1 Department of Medicinal Chemistry and Natural Medicine Chemistry, Department of Pharmacognosy, College of Pharmacy, Harbin Medical University, Harbin, 150081 China; 2 State-Province Key Laboratory of Biomedicine-Pharmaceutics of China, Harbin Medical University, Harbin, 150081 China; 3 School of Chinese Medicine, State Key Laboratory of Environmental and Biological Analysis, Hong Kong Traditional Chinese Medicine Phenome Research Center, Hong Kong Baptist University, Hong Kong, 999077 China; 4 Key Laboratory of Systems Biomedicine (Ministry of Education), Shanghai Center for Systems Biomedicine, Shanghai Jiao Tong University, Shanghai, 200240 China

**Keywords:** Zhishi Xiebai Guizhi Decoction, Pulmonary hypertension, Pulmonary vascular remodeling, Chemical compounds, HIF-1α

## Abstract

**Background:**

Pulmonary hypertension (PH) is a severe cardio-pulmonary vascular disease, involves complex molecular mechanism especially during the pathological process of pulmonary vascular remodeling, brings a significant challenge to clinical treatment and thus resulting in high mortality rates. Classic Traditional Chinese medicine formula, Zhishi Xiebai Guizhi Decoction (ZXGD), holds therapeutic potential for PH. In present study, we sought to explore therapeutic potential of ZXGD against PH in rats.

**Methods:**

We employed a combination methods of chemical profiling, echocardiographic, morphologic measurements, molecular biology, rats models and cultured pulmonary artery smooth muscle cells (PASMCs) to achieve this.

**Results:**

Eighteen compounds were precisely identified in ZXGD using UHPLC-QTOF-MS/MS. Our data demonstrated ZXGD could alleviate PH by reducing pulmonary artery pressure and alleviating pulmonary vascular remodeling in rats. Specifically, ZXGD was found to intervene in abnormal expansion of PASMCs, thereby attenuating pulmonary vascular remodeling. ZXGD was also observed to modulate expressions of HIF-1α, ROS, and Nrf2 to alleviate hypoxia and oxidative stress. Additionally, ZXGD significantly regulated disorders in pro-inflammatory cytokines, thus mitigating inflammation. Furthermore, ZXGD decreased levels of decadienyl-l-carnitine and LDL-C, while elevating HDL-C and lipid droplet counts, thereby reducing cholesterol and lipid toxicity and preserving mitochondrial function. Importantly, inhibition of HIF-1α reversed expression of key pathological triggers for pulmonary vascular remodeling. Neohesperidin and naringin in ZXGD extract were identified as the primary contributors to its pharmacological effects against PH.

**Conclusion:**

Altogether, our study empirically explored therapeutic potential and pharmacological mechanisms of ZXGD in treating PH, offering a groundwork for the development of novel anti-PH drugs.

**Graphical Abstract:**

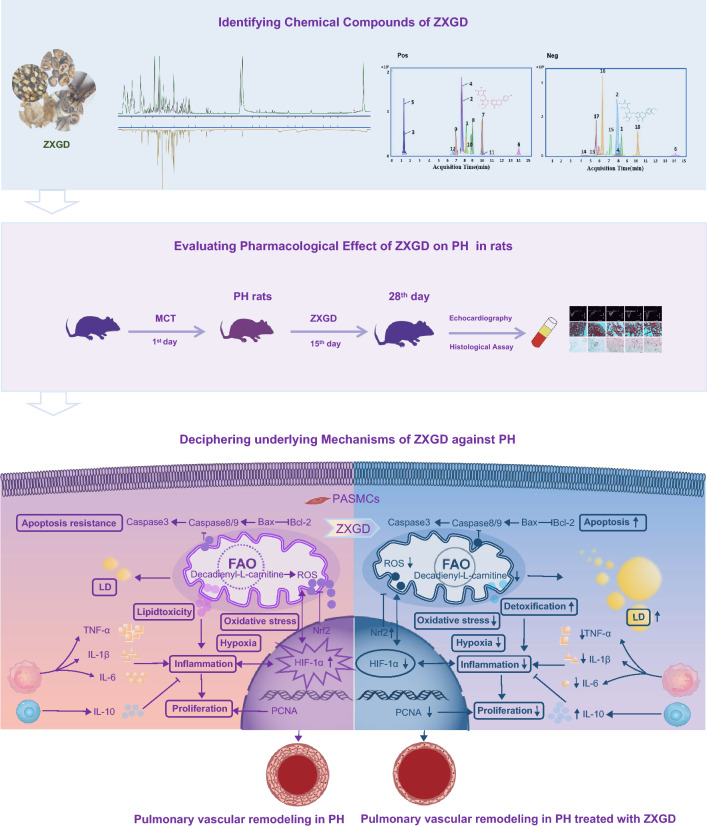

**Supplementary Information:**

The online version contains supplementary material available at 10.1186/s13020-024-01039-0.

## Introduction

Pulmonary hypertension (PH) is a lethal cardio-pulmonary vascular disease characterized by elevation of pulmonary arterial pressure, finally resulting in right heart failure or death [1, 2]. It affects approximately 1% of global population [3, 4], the median survival is only 2.8 years due to delayed diagnosis and lack of curative drugs [5]. PH is a complex and multiple factors disease, and classified into five groups [6]. Currently, there are no proven effective drugs for the treatment of patients with subtype II and III of PH correlated with left heart and lung disease [7]. One of the main challenges in existing drug treatments is that mono-target drugs are insufficient to address multiple targets to systematically restore the balance of molecular network involved in this complex disease. Despite multiple drug combinations could relieve symptoms and delay clinical worsening, PH remains associated with high morbidity and mortality [8]. Therefore, it is urgently need to develop novel therapeutic drugs with multiple-target characteristics for the treatment of PH [9].

One potential therapeutic option for clinical treatment of PH is the use of classical TCM formula with multiple ingredients and multiple targets [10–13]. Zhishi Xiebai Guizhi Decoction (ZXGD), firstly documented in Zhang Zhongjing’s Synopsis of Golden Chamber (Jin Kui Yao Lue in Chinese) in the Han Dynasty, is a well-known TCM classic formula in therapy for “obstructing chest” nearly two thousand years in clinic [14]. It consists of five Chinese herbal medicines, Trichosanthis Fructus (GuaLou), Allii Macrostemonis Bulbus (XieBai), Aurantii Fructus Immaturus (ZhiShi), Magnoliae Officinalis Cortex (HouPo), and Cinnamomi Ramulus (GuiZhi) [15]. ZXGD has shown significant alleviation effect in clinical symptoms of patients with cardiovascular diseases such as coronary heart disease (CHD), myocardial infarction, chronic heart failure, pulmonary thromboembolism [16, 17]. Moreover, Trichosanthis Fructus (Gualou) has been used to clear heat, remove phlegm, invigorate Qi, and eliminate chest congestion in TCM, and in therapy for cardiac failure, myocardial infarction, pulmonary heart disease in modern clinic [18]. Allii Macrostemonis Bulbus (Xiebai) has the function of activating Yang and smoothing Qi, removing stasis and alleviating stagnation, is often used for the treatment of CHD, angina pectoris, asthma, and diarrhea [19]. *Magnoliae officinalis* cortex (Houpo)’s benefit was for lung disease, and intestinal function [20]. ZXGD has been demonstrated a diversity of pharmacological activities, such as anti-inflammation, hypo-lipidemia, anti-hypoxia, anti-oxidation, anti-apoptosis, anti-coagulation, anti-fibrosis, anti-atherosis, improvement of blood rheology and oxygen partial pressure and regulation of neurotransmitters [14–16]. Furthermore, modern pharmacological studies indicated that bioactive components of herbal medicine in ZXGD have protective effects on experimental PH, such as Quercetin, Hesperetin, Luteolin, Osthole, naringenin et al. [21–30]. Despite abundant evidences were reported about its effectiveness, none experimental study confirms ZXGD could reduce pulmonary arterial pressure in modern pharmacology, the molecular mechanism of ZXGD against PH has not been fully investigated. Therefore, constituents identification, determination of therapeutic efficacy, discovery of crucial therapeutic targets, and elucidation of systematic therapeutic mechanisms of ZXGD are necessary and valuable to facilitate novel drug discovery against PH.

Pathological mechanism of PH is very complex, pulmonary vascular remodeling is critical pathological hallmark and crucial for PH treatment, manifested with thickening of medial vessel due to loss of pulmonary vascular homeostasis, driven by apoptosis inhibition and hyperproliferation of pulmonary artery smooth muscle cells (PASMCs), contributing to increase arterial resistance and pressure. To meet high energy demand of uncontrolled expansion of PASMCs, oxygen is fundamental substrate urgent in demands. Molecular oxygen is not only capable of generating cellular energy, but also produces toxic metabolite reactive oxygen species (ROS) from mitochondria [31]. Decreased oxygen availability could severely damage mitochondria function, up-regulate HIF-1α expression and ROS signaling, impair lipid metabolism [24, 32–34]. HIF-1α overexpression, ROS and pro-inflammatory lipids accumulation induce inflammation and disrupt vascular cell function, which is an early hallmark and primary pathological feature that initiates and accelerates the development of pulmonary vascular remodeling [35]. Therefore, hypoxia, oxidative stress, inflammation, mitochondrial dysfunction, lipid metabolic dysregulation are crucial pathogenic trigger factors for pulmonary vascular remodeling. HIF-1α, as a pivotal regulator response to oxygen homeostasis, metabolism, inflammation and in crosstalk with mitochondria, is the underlying mechanism of determining disease progression in PH [36–39].

ZXGD has been used for the treatment of “obstructing chest” caused by “Yang deficiency” and “phlegm stagnation” in TCM. The essence of “Yang deficiency” in whole body is mitochondrial dysfunction, and connected with hypoxia [40]. Phlegm stagnation is associated with hyperlipidemia in modern medicine, blocks arteries and veins of heart by high levels of low-density lipoprotein cholesterol (LDL-C), total cholesterol (TC) and low concentration of high-density lipoprotein cholesterol (HDL-C) [41].

Accordingly, ZXGD shows therapeutic potential for PH. This study aimed to investigate pharmacological effects, functional compounds, and molecular mechanisms of ZXGD on PH, providing a molecular basis for the development of novel drugs for PH treatment.

## Materials and methods

### Chemicals and reagents

Monocrotaline (MCT, 95%) was purchased from Aladdin Co. (Shanghai, China). Methanol, isopropanol, and formic acid (LC/MS grade) were from Macklin Biochemical Co., Ltd (Shanghai, China). Acetonitrile was from Adamas-beta (Shanghai, China). Neohesperidin, Hesperitin, Scopoletin, Esculetin, Neoeriocitrin, Lonicerin, Naringin, Naringenin, Limonin, Adenosine, Adenine, Syringic acid, Magnoloside A and Magnoloside B, Isosakuranetin, Xanthotoxol, Eriocitrin, Poncirin were from Sigma-Aldrich (Shanghai, China), Yangling Ciyuan Biotech Co., Ltd. (Shaanxi, China), Baoji Fang Sheng biological development Co., Ltd. (Shaanxi, China), Shanghai Sunny Biotech Co., Ltd. (Shanghai, China), Shanghai Standard Technology Co., Ltd. (Shanghai, China). The purity of these reference compounds were over 98%.

### Preparation of ZXGD

Chinese herbal medicine in ZXGD were purchased from RenminTongtai Pharmacy (Harbin, China). According to the decocting method recorded in the Synopsis of Golden Chamber, 240g of Aurantii Fructus Immaturus and 240 g of Magnoliae Officinalis Cortex and 4000 ml distilled water were decocted to 1600 ml. After filtration, 480 g of Trichosanthis Fructus, 180 g of Allii Macrostemonis Bulbus, and 60 g of Cinnamomi Ramulus were then added into the filtration for another decocting and condensed into 1 g/ml. Finally, the decoction was filtered and freeze-dried.

### Chemical characterization of ZXGD

A UHPLC system (Agilent 1290 Infinity, Agilent Technologies, USA) integrated with QTOF-MS (Agilent 6560 IM-QTOF, Agilent Technologies, USA) were used to analyze the chemical compounds of ZXGD. An ACQUITY UPLC®BEH C18 column (2.1 × 100 mm, 1.7 μm, Waters) was applied for chromatographic separation of ZXGD. The mobile phase is composed of 0.1% formic acid with water (A) and 0.1% formic acid with acetonitrile (B). We provided the QTOF parameters in Supplementary Table 1. The information of 18 precisely identified compounds of ZXGD are listed in Supplementary Table 2.

### Animals

Adult Sparague Dawley rats (male, weight of 200 ± 20 g) were obtained from the Experimental Animal Center of Harbin Medical University. All animal experiments methods in present study were carried out with the approval of the Ethical Committee of Laboratory Animals at Harbin Medical University (No. IRB3011621), Harbin, China, and with the conformity to the Chinese National Regulations on the Use of Experimental Animals.

We used PH rats induced with MCT because it is the most classical and widely used in vivo model for nearly 60 years due to its simplicity, reproducibility and low cost [42]. The following animal experiments were conducted: Rats were randomly assigned into five groups (n = 10, respectively), PH group, 2.7 g/kg ZXGD treatment group, 5.4 g/kg ZXGD treatment group, and 35 mg/kg Sildenafil treatment group rats were firstly intraperitoneally injected with 60 mg/kg MCT to induce PH occurrence for 28 days. Control group rats were received the same volume of saline as MCT. On the 15th day after MCT post-injection, rats in ZXGD treatment groups and Sildenafil group were orally administered with different dosage of ZXGD and 35 mg/kg Sildenafil daily for consecutive 14 days.

### Echocardiography

All experimental rats were received a single intraperitoneal injection with chloral hydrated (300 mg/kg) to anesthetize to be prepared for echocardiographic examination. Echocardiographic assessment of pulmonary vascular function in rats was performed with a digital imaging platform coupled with linear array technology and Doppler mode (Vevo 2100, Visual Sonics, Toronto, Canada). Echocardiographic images were collected with pulsed wave Doppler, hemodynamic measurements of pulmonary blood flow including pulmonary arterial acceleration time (PAT), ejection time (ET), and PAT/ET were regarded as alternative indexes to estimate RVSP.

### Histological assay

Using 4% paraformaldehyde, graduated alcohol and paraffin wax, the lung leaflets were received a fixation for overnight, then dehydration, subsequent embedding in paraffin wax. Further staining was performed with hematoxylin and eosin (H&E). The tissue pieces for immunohistochemistry were deparaffinized and rehydrated, then incubated with α smooth muscle actin (α-SMA) antibody (Boster, Wuhan, China) for overnight, subsequent 2 h incubation with goat anti-mice IgG. Brown color indicated positive stains. H&E and immunohistochemistry were visualized with an Eclipse 600 Nikon microscope and photographed with a digital camera, then analyzed using Image Pro Plus software. Measurement of diameter and area of each pulmonary microartery, then calculated wall thickness and positive stain area of α-SMA of pulmonary vascular.

### Western blot

The methods in extraction, separation, and transferring membranes of protein samples from lung tissues and PASMCs were performed according to our previous study [24], further incubation with the following primary antibodies: Caspase 3, Caspase 9, PLA2, PPARγ, IL-1β, IL-10 (Bioss, Beijing, China); Bax, HIF-1α, PLIN2 (Boster, Wuhan, China); PCNA (Wanleibio, Shenyang, China); IL-6 (Beyotime, Shanghai, China); Caspase 8, Bcl-2, β-actin (ABclonal, Wuhan, China) for overnight at 4 °C and subsequently incubated with secondary antibodies (goat anti-rabbit IgG, Bioss, Beijing, China) for 1h at room temperature. Immunoblots were scanned with Odyssey clx. The relative expression of protein to the control β-actin was determined by densitometric analysis using Quantity one software.

### Enzyme-linked immunosorbent assay and biochemical analysis

Rat ELISA kits (MEIMIAN, Yancheng, China) were used to test the levels of decadienyl-l-carnitine, IL-1β, IL-6, IL-10, and TNF-α in lung tissue. Biochemical assay kits were employed to examine the serum concentration of TC, LDL-C and HDL-C in rats (Nanjing Jiancheng Bioengineering Institute, China). Measurement of Optical density (O.D) and calculation of concentration of each sample are based on a Microplate reader (Biotek, USA) and standard curve linear regression equation.

### Cell culture

Acquisition and culture of PASMCs have been reported in our previous study [43]. Platelet-derived growth factor (PDGF)-BB was used to mediate PASMCs proliferation in vitro [44]. Before each experiment, the cells were subjected to growth arrest for 24 h, then exposed to different dosage of ZXGD, neohesperidin and naringin treated with or without 40 ng/ml PDGF-BB according to the experimental requirements.

### MTT assay

PASMCs were exposed to different dosage of ZXGD, neohesperidin, and naringin with or without PDGF-BB treatment for 24 h incubation at 37 °C, then subjected to 20% FBS-DMEM medium including MTT reagent (Beyotime, Shanghai, China) incubation for 4 h, and blocked with DMSO. Subsequently, the samples were examined using a Microplate reader at 490 nm.

### Oil red O staining

According to different experimental requirements, PASMCs were treated with 40 ng/ml PDGF-BB, 5 mg/ml and 10 mg/ml ZXGD, then incubated for 24 h. Next, incubated with 60% isopropanol for 5 min, rinsed twice with distilled water, subsequently oil red O dyeing solution (Beyotime, Shanghai, China) was added for 20–30 min, then hematoxylin dyeing for 1 min. Finally, microscope and Image Pro Plus software were applied for observation, visualization and analysis of PASMCs.

### Measurement of ROS

PASMCs were exposed to 40 ng/ml PDGF-BB and different dosage of ZXGD (5 mg/ml, 10 mg/ml) for 24 h, then DCFH-DA probe (10 μM) (ROS Assay Kit, Beyotime, Shanghai, China) for 30 min at 37 ℃. CytoFLEX flow cytometry (FCM, Beckman Coulter, USA) and FlowJo 10.8.1 software were applied in this study for the detection and analysis of ROS.

### Transient transfection of HIF-1α

The primer sequence of HIF-1α siRNA (GenePharma, Shanghai, China) and negative control (NC) were used as follow: HIF-1α siRNA sense 5′-CGCAUUGAA GUUAGAGUCAATT-3′ and antisense 5′-UUGACU CUAACUUCAAUGCTT-3′; NC sense 5′-UUCUCCGAACGUGUCACGUTT-3′ and antisense 5′-ACGUGACACGUUCGGAGAATT-3′. PASMCs cells were transfected with HIF-1α siRNA containing GP-transfect-Mate according to the manufacture’s instructions.

### qRT-PCR

Total RNA was extracted from lung tissues and PASMCs with Trizol reagent (Beyotime Biotechnology, China) and reverse-transcribed using BeyoRT™II First Strand cDNA Synthesis Kit (Beyotime, Shanghai, China). The primer sequences of HIF-1α and β-actin were used as follows: HIF-1α forward 5′ TGGACTTGCCCC TTTCTCTG-3′ and reverse 5′-CGACGTTCGGAACTCATCCT-3; β-actin forward 5′-AAGTTCTACAAATGTGGC TGAGGA-3′ and reverse 5′-TCCTCTTAGGAGTGGG GGTGG-3′. Then, PCR was conducted using SYBR Green (Thermo, USA). The relative level of HIF-1α was calculated by the comparative Cq method (2−^ΔΔCT^).

### Statistical analysis

One-way analysis of variance (ANOVA) is performed for statistical analysis using GraphPad Prism 5.0. All values were presented as mean ± SEM. *p* < 0.05 was set as statistical significance.

## Results

### Identification of main compounds in ZXGD with LC–MS/MS based chemical profiling

**Fig. 1:**
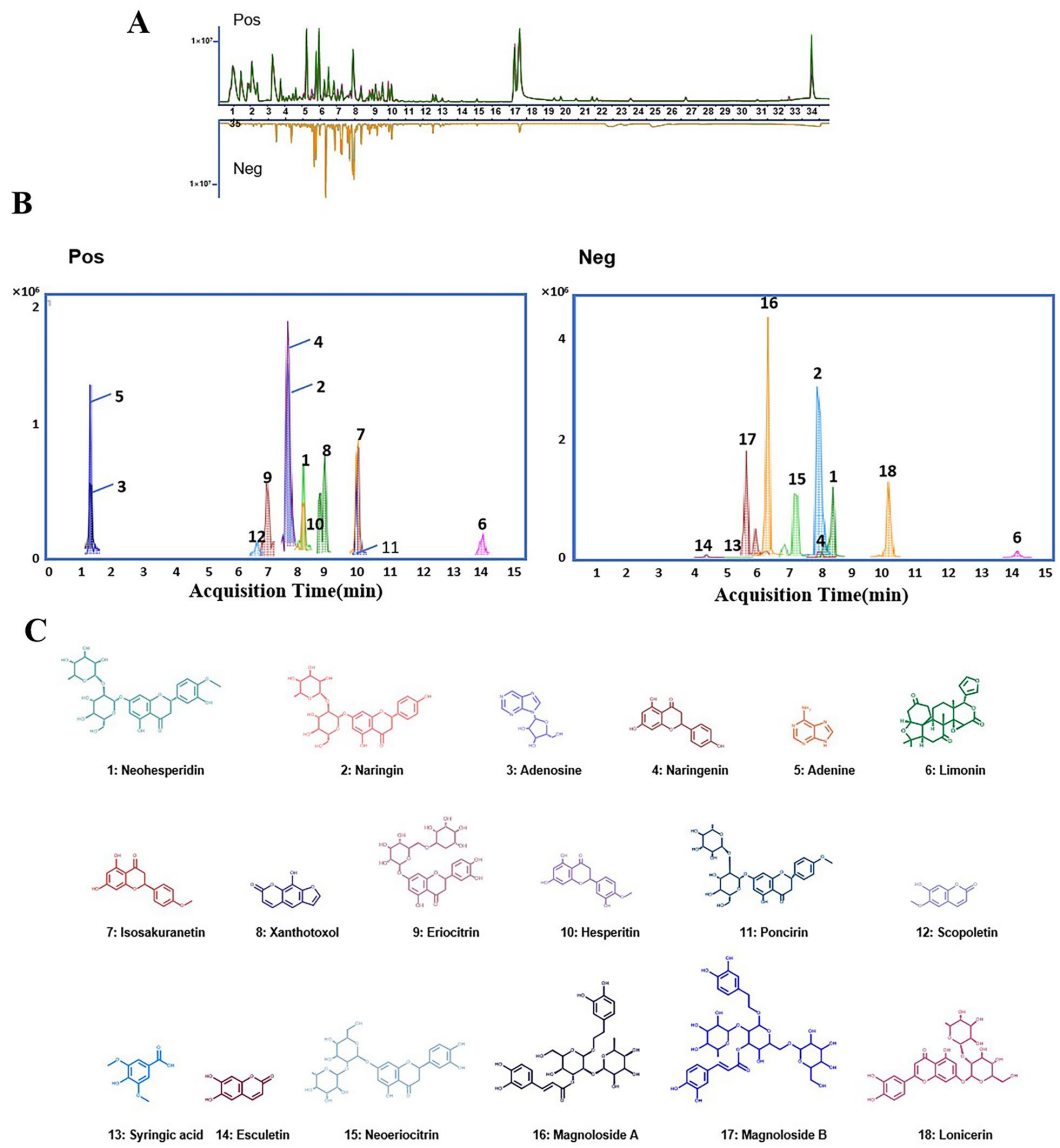
Mass spectrometry based chemical profiling method precisely identified 18 main compounds in ZXGD extract. **A** The base peak chromatogram (BPC) of ZXGD based on UHPLC-TOF–MS. **B** The extracted ion chromatograms (EICs) of 18 chemical components in ZXGD in both positive and negative ion modes. **C** Peaks 1–18 were identified and assigned as neohesperidin, naringin, adenosine, naringenin, adenine, limonin, isosakuranetin, xanthotoxol, eriocitrin, hesperitin, poncirin, scopoletin, syringic acid, esculetin, neoeriocitrin, magnoloside a, magnoloside b, lonicerin

To assess therapeutic effectiveness and mechanism of ZXGD in treating PH, it is imperative to identify and characterize key chemical compounds presented in ZXGD. To accomplish this, a highly sensitive UHPLC-QTOF-MS/MS-based chemical profiling method was utilized to accurately pinpoint the major compounds in ZXGD extract. A total of 18 primary compounds were detected and precisely identified in ZXGD extract by precisely matching MS/MS spectra of compound features with known reference compounds. These compounds include neohesperidin, naringin, adenosine, naringenin, adenine, limonin, isosakuranetin, xanthotoxol, eriocitrin, hesperitin, poncirin, scopoletin, syringic acid, esculetin, neoeriocitrin, magnoloside a, magnoloside b, and lonicerin (Fig. 1). In essence, this data provides a crucial chemical basis for further unraveling primary bioactive compounds presented in ZXGD that may be beneficial for combating PH.

### ZXGD reversed apoptosis resistance and augmented proliferation of PASMCs in vitro

**Fig. 2:**
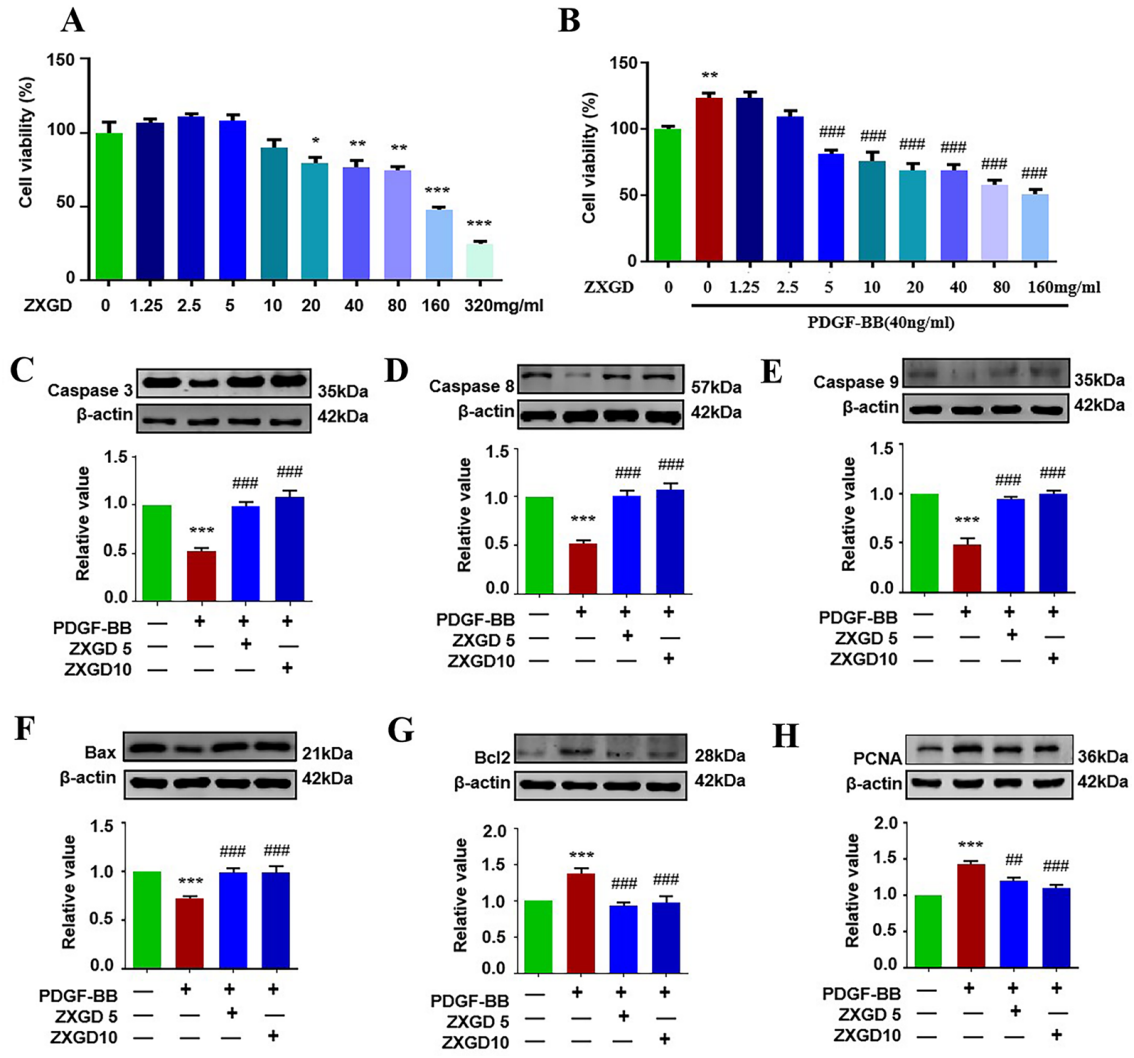
ZXGD modulated the apoptosis resistance and augmented proliferation of PASMCs in vitro*.* **A**, **B** Effects of ZXGD on viability of PASMCs treated with and without PDGF-BB were examined by MTT (n = 6). **C**–**H** Expression levels of Caspase 3, Caspase 8, Caspase 9, Bax, Bcl2 and PCNA in PASMCs (n = 6). **p < 0.01, ***p < 0.001 vs control, ^##^*p* < 0.01, ^###^*p* < 0.001 vs PDGF-BB. 5 represents 5 mg/ml, 10 represents 10 mg/ml

To investigate pharmacological activity of ZXGD on PASMCs so as to explore its therapeutic potential on pulmonary vascular remodeling, we detected effect of ZXGD on PASMCs exposed to PDGF-BB by examining cell viability, proliferation and apoptotic proteins. As shown in Fig. 2A and B, the optimal dosage of ZXGD in suppressing PASMCs proliferation was determined as 5 mg/ml and 10 mg/ml, therefore 5 mg/ml and 10 mg/ml ZXGD were used in following vitro study. In addition, there was a significant down-regulation in apoptosis initiator caspase 8 and caspase 9, apoptosis effector caspase 3, and apoptosis promotion protein Bax, but up-regulation in apoptosis resistance protein Bcl2 and proliferation protein proliferating cell nuclear antigen (PCNA) in PDGF-BB induced PASMCs group when compared to control group. After administrated with ZXGD, these proteins expression were largely reversed to normal level. These findings demonstrated that ZXGD can reverse augmented proliferation and apoptosis resistance of PASMCs through modulating caspase 3, caspase 8, caspase 9, Bax, Bcl 2 and PCNA in vitro, indicated the therapeutic potential of ZXGD for alleviating pulmonary vascular remodeling.

### ZXGD significantly alleviated pulmonary arterial pressure and attenuated pulmonary vascular remodeling in rats with PH

**Fig. 3:**
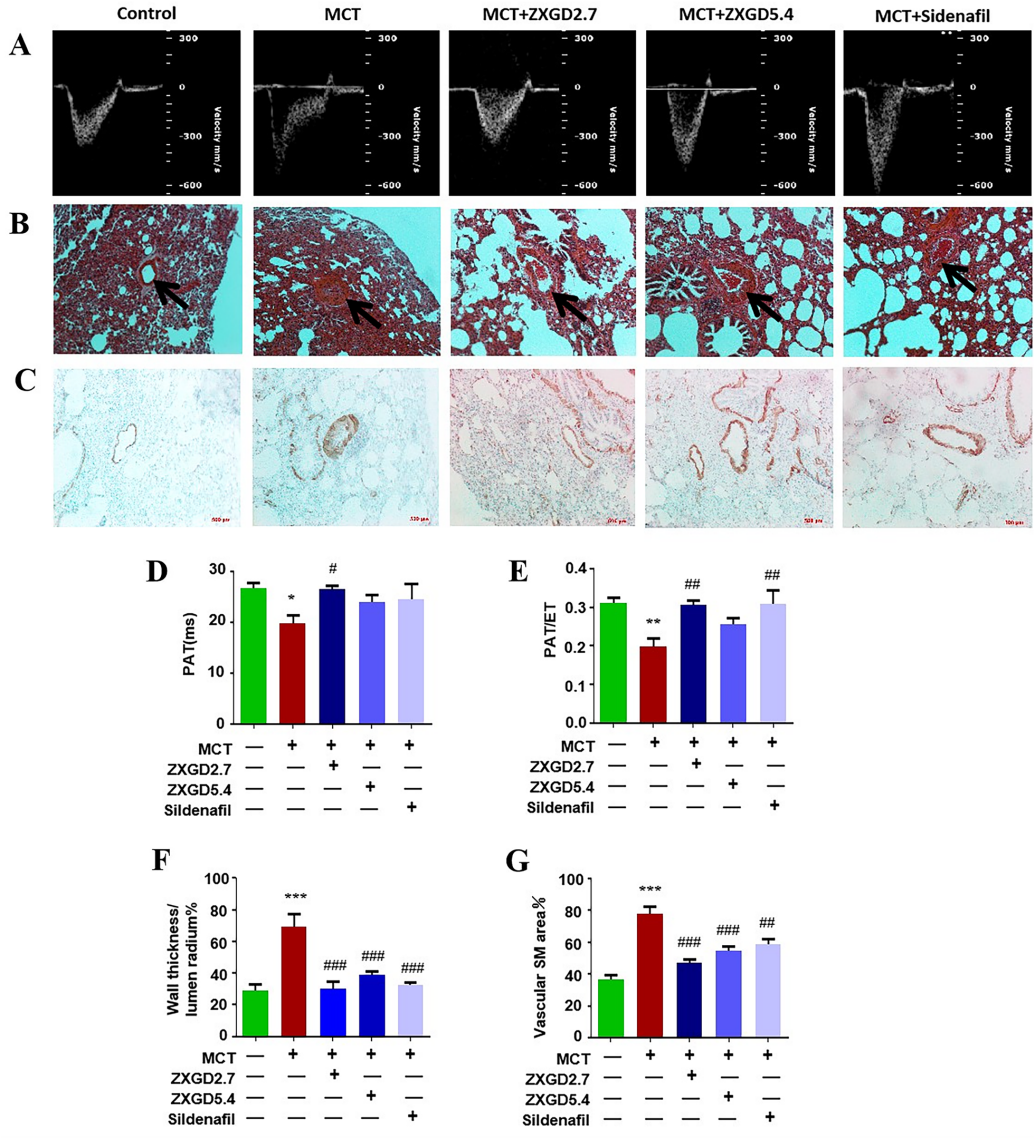
ZXGD alleviated pulmonary arterial hypertension and inhibited pulmonary vascular remodeling in MCT induced PH rats. **A** Representative images of pulmonary flow were measured by pulsed-wave Doppler. **D**, **E** Statistical analysis of pulmonary acceleration time (PAT) and the ratio PAT to right ventricular ejection time (ET) (PAT/ET) (n = 4). **B**, **C** HE and α-SMA staining of pulmonary vessels in rats (magnification, ×200). **F**, **G** Quantitative analysis of wall thickness and positive staining of pulmonary vessels (n = 4). *p < 0.05, **p < 0.01, ***p < 0.001 vs control; ^#^*p* < 0.05, ^##^*p* < 0.01, ^###^*p* < 0.001 vs PH. 2.7 represents 2.7 g/kg, 5.4 represents 5.4 g/kg

To explore therapeutic capacity of ZXGD against PH in vivo, we first assessed phenotypic index pulmonary arterial pressure in rats using noninvasive echo Doppler. The ratio of pulmonary arterial acceleration time (PAT) to ejection time (ET) as well as PAT are representative indexes of right ventricular systolic pressure (RVSP) [45]. As shown in Fig. 3, pulmonary artery pressure was significantly increased in rats with PH, characterized by a shortened PAT and a decreased PAT/ET ratio. However, ZXGD was able to reverse these changes and restore pulmonary arterial pressure to normal levels, significantly in 2.7 g/kg ZXGD, indicated ZXGD effectively decreased pulmonary arterial pressure.

Next, we performed HE and α-SMA staining to observe wall thickness and muscularization of pulmonary arteries thus to determine pulmonary vascular remodeling. Compared to control group, PH group showed increased thickness of pulmonary vascular wall, particularly in medial smooth muscle layer (Fig. 3B). Additionally, there was a significant increase in α-SMA staining, indicating PASMCs proliferation and pulmonary vascular muscularization (Fig. 3C). However, different dosages of ZXGD treatment both effectively ameliorated these changes, demonstrating the ability of ZXGD to inhibit pulmonary vascular remodeling.

### ZXGD ameliorated pulmonary vascular remodeling by modulating protein expression of proliferation and apoptosis in vivo

**Fig. 4:**
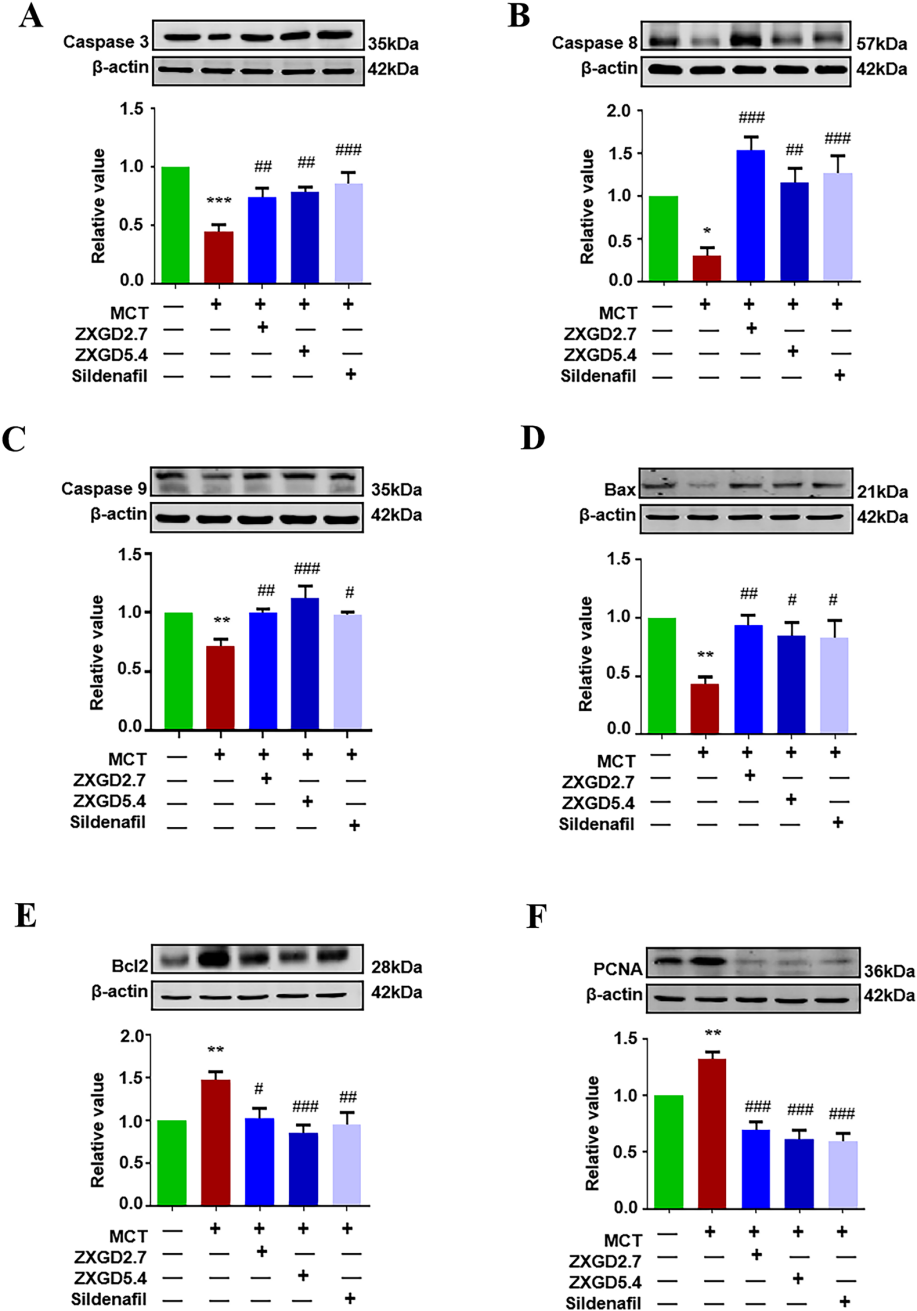
ZXGD ameliorated pulmonary vascular remodeling by modulating the proliferation and apoptosis proteins expression in vivo. **A**–**F** Expression levels of Caspase 3, Caspase 8, Caspase 9, Bax, Bcl2 and PCNA in lung tissues(n = 6). *p < 0.05, **p < 0.01, ***p < 0.001 vs control, ^#^*p* < 0.05, ^##^*p* < 0.01, ^###^*p* < 0.001 vs PH

To further determine pharmacological mechanism of ZXGD against pulmonary vascular remodeling in vivo, we investigated effect of ZXGD on proliferation and apoptotic proteins in lung tissue. As shown in Fig. 4, consistent with the results in PASMCs, ZXGD administration could markedly increase the expression of pro-apoptotic proteins Bax, caspase 3, caspase 8, caspase 9, and significantly decrease the expression of anti-apoptotic protein Bcl2 and proliferation related protein PCNA when compared with PH group. These findings demonstrated that ZXGD may modulate the balance between proliferation and apoptosis proteins to control PASMCs expansion, leading to change in pulmonary vascular structure, thus to alleviate pulmonary vascular remodeling.

### ZXGD modulated hypoxia, oxidative stress and inflammation to ameliorate pulmonary vascular remodeling

**Fig. 5:**
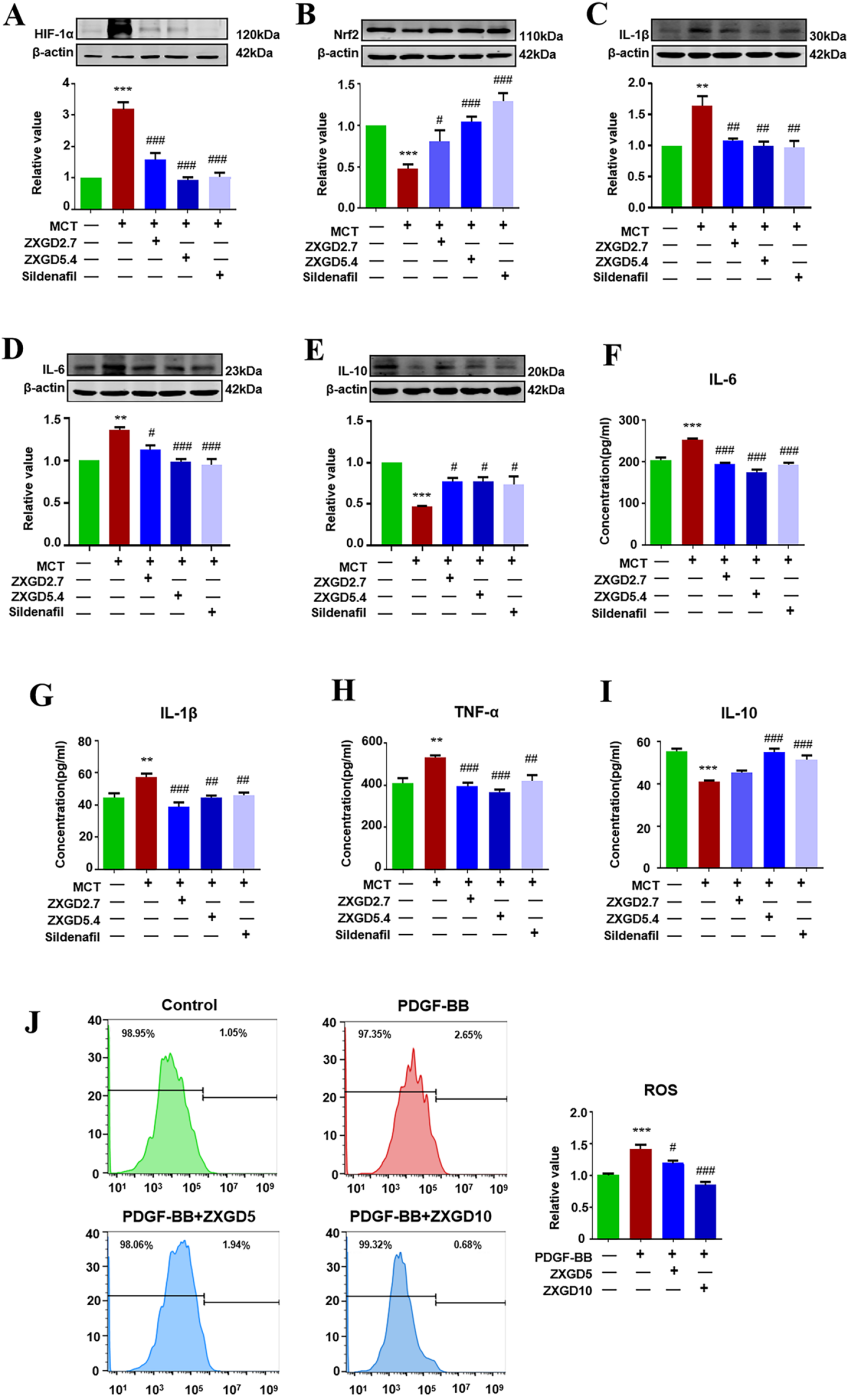
ZXGD inhibited hypoxia, oxidative stress and inflammation in rats with PH and PASMCs induced with PDGF-BB. **A**–**E** Expression of HIF-1α, Nrf2, IL-1β, IL-6 and IL-10 in lung tissue of rats were detected using Western blot (n = 3 or 6). **F**–**I** Concentration of IL-6, IL-1β, TNF-α and IL-10 in lung tissue of rats were examined by ELISA (n = 6). **J** Level of ROS in PASMCs was assessed with flow cytometry, and statistical data was obtained. **p < 0.01, ***p < 0.001 vs control, ^#^*p* < 0.05, ^##^*p* < 0.01, ^###^*p* < 0.001 vs PH or PDGF-BB

To further investigate effects of ZXGD on triggers for pulmonary vascular remodeling, such as hypoxia, oxidative stress, and inflammation, we conducted several tests. HIF 1-α, anti-oxidative stress protein Nuclear factor E2-related factor 2 (Nrf2), IL-6, IL-1β, TNF-α, and IL-10 in lung tissues were measured by Western blot and ELISA, ROS in PASMCs was assessed using flow cytometry. As shown in Fig. 5, our results demonstrated PH group exhibited significantly high levels of HIF-1α and pro-inflammatory cytokines IL-6, IL-1β, TNF-α, and low levels of Nrf2 and anti-inflammatory cytokine IL-10 compared to control group. Additionally, ROS production was increased in PASMCs exposed to PDGF-BB. However, after treatment with ZXGD, these cytokines and proteins returned to normal levels, similar to that in control group. These findings uncovered that ZXGD can relieve hypoxia and oxidative stress, and inhibit inflammation in rats with PH. It achieves this by restoring pulmonary vascular homeostasis through promoting beneficial factors and blocking adverse factors associated with pulmonary vascular remodeling.

### ZXGD regulated lipid metabolism to reduce cell lipid toxicity in rats with PH

**Fig. 6:**
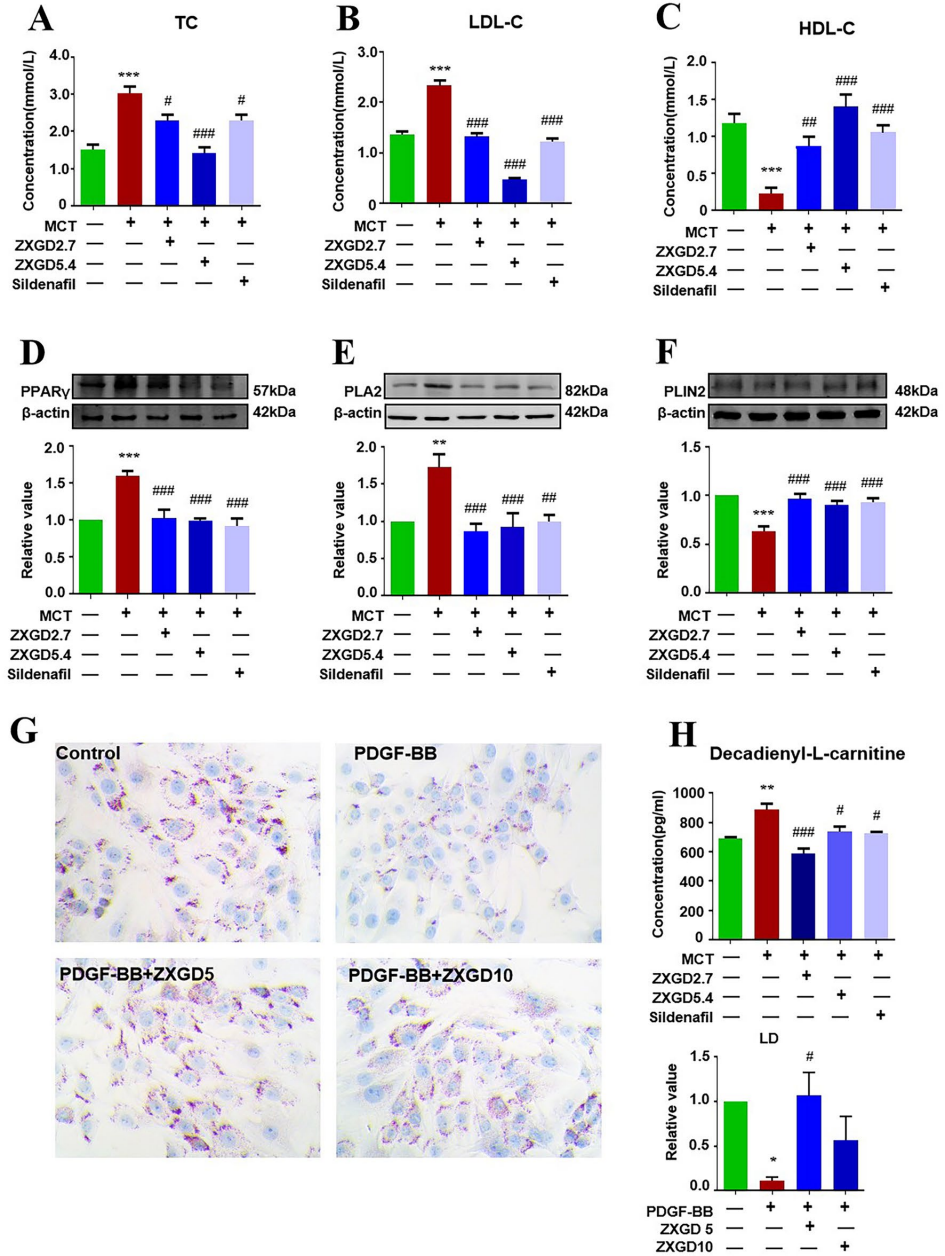
ZXGD regulated lipid metabolism to reduce cell lipid toxicity in rats with PH. **A**–**C**, **H** Concentration of TC, LDL-C and HDL-C (n = 6) in serum as well as decadienyl-l-carnitine (n = 3) in lung tissue were examined by ELISA and Biochemical kit. **D**–**F** Expression levels of PPARγ, PLA2 and PLIN2 in lung tissue were detected using Western blot (n = 6). **G** Count of lipid droplets in PASMCs was assessed with Oil red O staining, and statistical data were obtained. *p < 0.05, **p < 0.01, ***p < 0.001 vs control, ^#^*p* < 0.05, ^##^*p* < 0.01, ^###^*p* < 0.001 vs PH or PDGF-BB

To further elucidate modulatory mechanism of ZXGD on lipid metabolism dysregulation involved in pulmonary vascular remodeling, firstly, we tested the levels of TC, LDL-C, and HDL-C in serum, additionally, we investigated the expression of key proteins involved in lipid metabolism, including peroxisome proliferator-activated receptor gamma (PPARγ), phospholipase A2 (PLA2), and the marker protein for lipid droplets perilipin 2 (PLIN2), furthermore, we assessed lipid metabolite decadienyl-l-carnitine (C10:2) in lung tissues, lastly, we analyzed lipid droplets in PASMCs using oil red O staining. As depicted in Fig. 6, concentration of TC and LDL-C in serum, as well as expression of PPARγ, PLA2, and decadienyl-l-carnitine in lung tissue, were significantly elevated in PH group compared to control group. Conversely, level of HDL-C, number of lipid droplets in PASMCs, and expression of PLIN2 were significantly reduced. These findings indicate that PH rats induced with MCT experienced high cholesterol level, excessive fatty acid oxidation, and cellular lipotoxicity. However, ZXGD treatment effectively reversed lipid metabolism dysregulation and restored lipid droplet counts, thereby protecting against cell lipidotoxicity. These results indicate that ZXGD has potential to down-regulate TC and LDL-C while up-regulate HDL-C in serum. Furthermore, ZXGD can inhibit fatty acid oxidation, promote lipid droplet formation, and enhance cholesterol esterification. Ultimately, these effects alleviate cell lipidotoxicity caused by decadienyl-l-carnitine and LDL-C.

### ZXGD inhibited HIF-1α-mediated pulmonary vascular remodeling

**Fig. 7:**
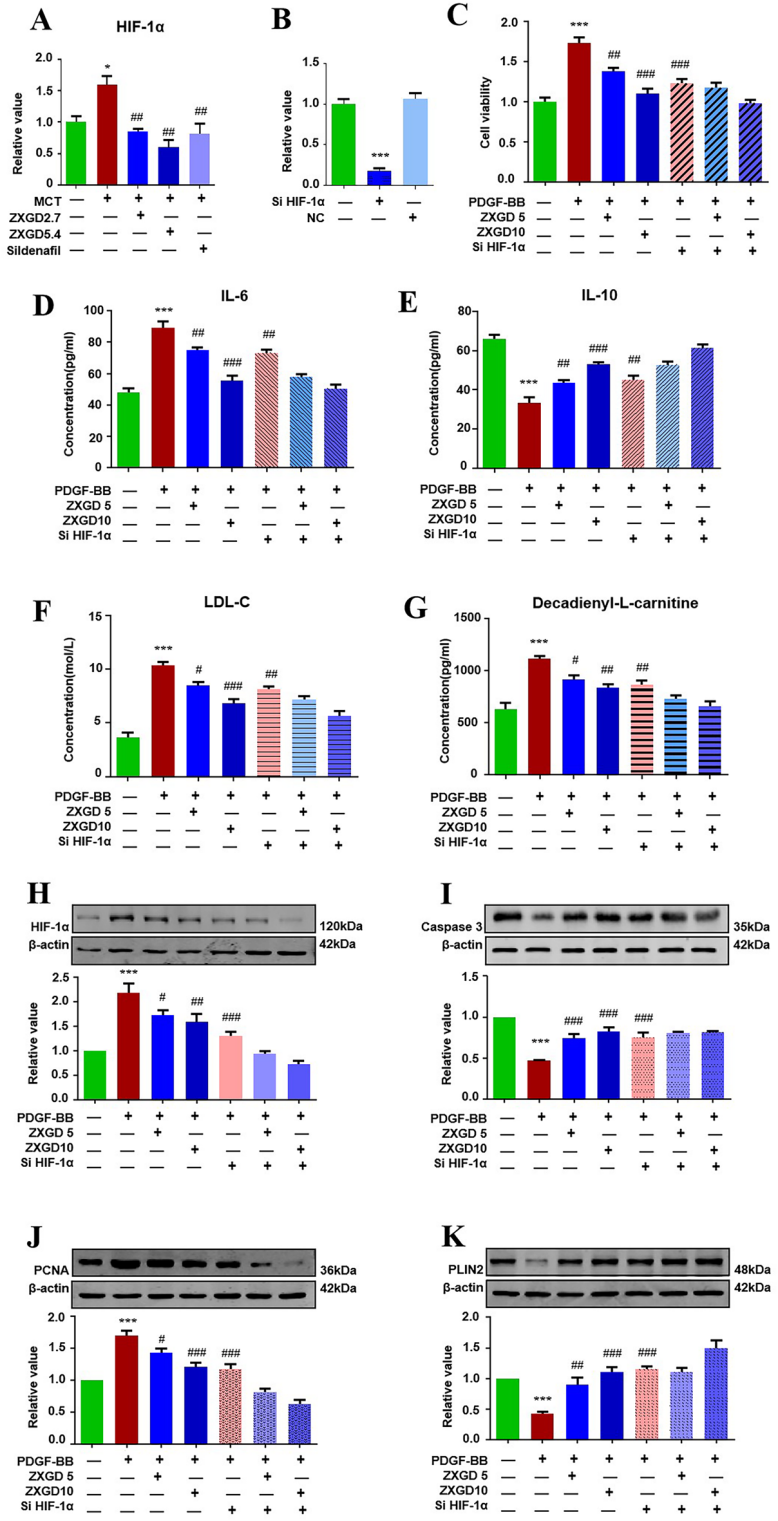
ZXGD modulated HIF-1α mediated pulmonary vascular remodeling. **A** Expression level of HIF-1α in lung tissue of rats was tested with PCR (n = 3). **B** Expression level of HIF-1α in PASMCs transfected with siRNA (n = 6). **C** Effects of ZXGD on viability of PASMCs transfected with HIF-1α siRNA was examined by MTT (n = 6). **D**–**G** Levels of IL-6, IL-10, LDL-C and decadienyl-l-carnitine in PASMCs transfected with HIF-1α siRNA. **H**–**K** Expression level of HIF-1α, Caspase-3, PCNA, PLIN2 in PASMCs transfected with HIF-1α siRNA (n ≥ 3). *p < 0.05, ***p < 0.001 vs control, ^#^*p* < 0.05, ^##^*p* < 0.01, ^###^*p* < 0.001 vs PH or PDGF-BB

To identify the key modulator in therapeutic targets network of ZXGD against pulmonary vascular remodeling, we first examined expression of HIF-1α gene in lung tissues, then silenced HIF-1α gene using siRNA transfection and evaluated the critical markers of proliferation, apoptosis, hypoxia, inflammation, cholesterol, lipid droplets, and lipid toxicity among therapeutic targets of ZXGD in vitro using MTT, PCR, ELISA, and Western blot assays. As shown in Fig. 7, expression level of HIF-1α gene was upregulated in rats with PH group when compared to that in control group. However, ZXGD administration and HIF-1α siRNA both obviously downregulated the gene expression of HIF-1α, indicating ZXGD and siRNA both effectively inhibited transcriptional regulation of HIF-1α in PH (Fig. 7A, B). After silencing HIF-1α gene, PASMCs viability, levels of LDL-C, IL-6, decadienyl-l-carnitine, as well as the expression of HIF-1α and PCNA were significantly suppressed, conversely, level of IL-10, expression of caspase 3 and PLIN2 were significantly increased in PASMCs exposed to PDGF-BB. Furthermore, the inhibitory effect of ZXGD was similar to siHIF-1α, suggesting HIF-1α, as the core target of ZXGD, has the capability to synergistically regulate multiple therapeutic targets involved in pathological trigger factors for pulmonary vascular remodeling.

### Neohesperidin and naringin exerted the same pharmacological effect as ZXGD on PASMCs

**Fig. 8:**
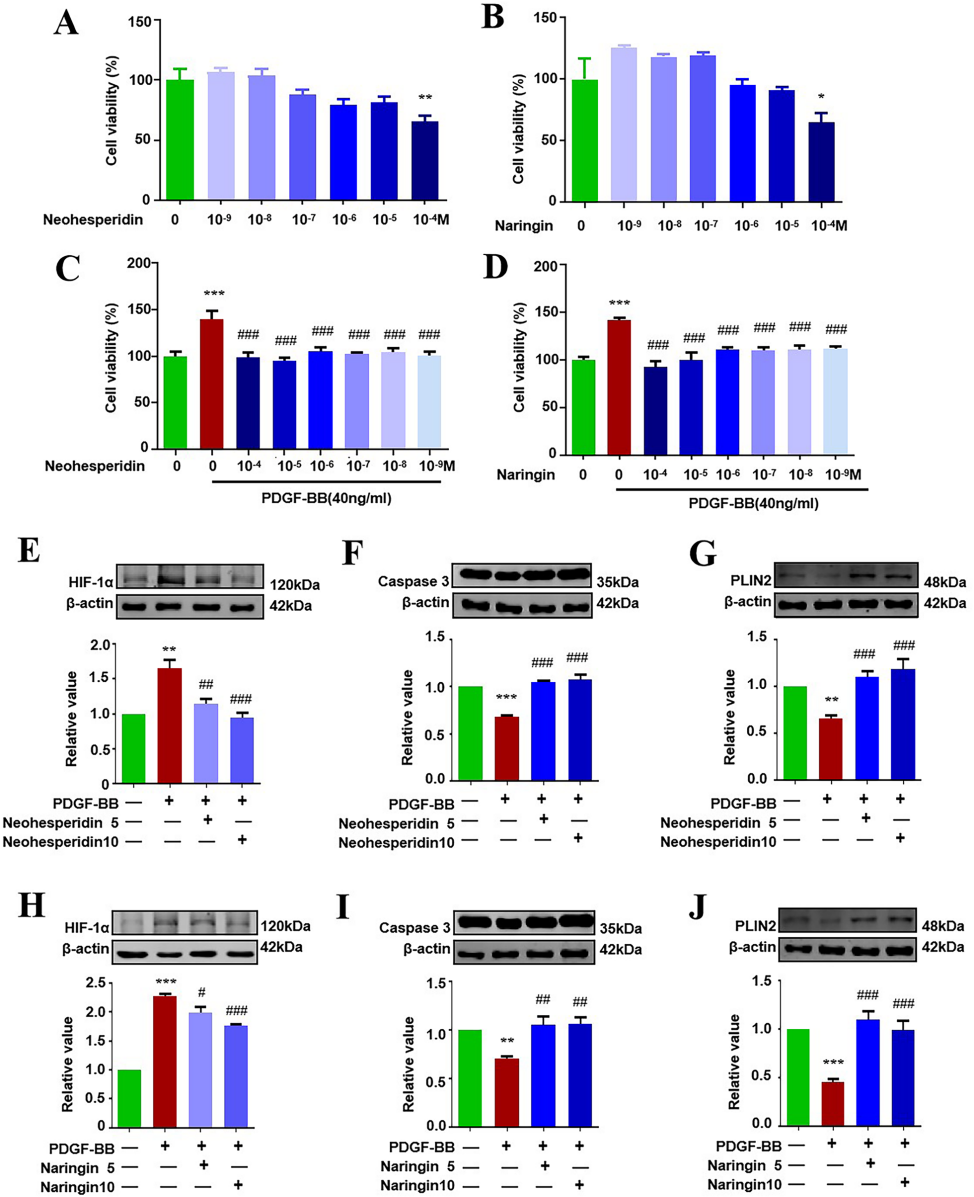
Effect of neohesperidin and naringin on cell viability, HIF-1α, Caspase3, PLIN2 in PASMCs. **A**–**D** Effects of neohesperidin and naringin on PASMCs viability were observed by MTT (n = 6). **E**–**J** Expression levels of HIF-1α, Caspase3, PLIN2 in PASMCs were evaluated by Western Blot (n ≥ 3). *p < 0.05, **p < 0.01, ***p < 0.001 vs control, ^#^*p* < 0.05, ^##^*p* < 0.01, ^###^*p* < 0.001 vs PDGF-BB. 5 represents 5 µM, 10 represents 10 µM

To further determine novel pharmacological effect of naringin and neohesperidin in ZXGD, we assessed their effects on PASMCs by measuring cell viability and key regulators in hypoxia, apoptosis, and lipid droplet counts. We first investigated effect of neohesperidin and naringin on PASMCs and found 10µM neohesperidin and 10µM naringin both markedly inhibited PASMCs proliferation (Fig. 8A–D). We then examined the expression levels of hypoxia modulator HIF-1α, apoptosis marker caspase 3, and lipid droplet marker PLIN2 in PASMCs treated with neohesperidin and naringin. Results showed that neohesperidin and naringin both significantly reversed the changes induced by PDGF-BB, restoring these markers to normal levels. These findings demonstrate that neohesperidin and naringin have a novel bioactivity of promoting apoptosis, ameliorating hypoxia, and upregulating lipid droplets to inhibit PASMCs proliferation, suggesting their potential in alleviating pulmonary vascular remodeling.

## Discussion

In present study, we are first time to investigate effect of ZXGD on experimental PH in rats. The major findings are (1) Eighteen compounds were precisely identified in ZXGD with a LC–MS/MS based chemical profiling method, neohesperidin and naringin have a potential in alleviating pulmonary vascular remodeling; (2) ZXGD administration effectively reverses the increase in pulmonary arterial pressure and alleviates pulmonary vascular remodeling in rats with PH; (3) ZXGD exerts multiple pharmacological effects to ameliorate pulmonary vascular remodeling targeting by HIF-1α thus to alleviated PH.

We were the first to precisely identify eighteen compounds in ZXGD with a LC–MS/MS based chemical profiling method. Among these compounds, magnoloside a was presented with highest abundance in ZXGD. Additionally, adenosine, as an important energy resource for ATP production, may play a role in addressing “Yang deficiency” in “obstructing chest” [46]. Nine of the identified compounds are flavonoids with strong cardiovascular bioactivity. Neohesperidin and naringin, derived from four herbs in ZXGD, have strong antioxidant, anti-inflammatory, and hypotensive effects [47, 48]. These two compounds have previously been identified as marker contents of ZXGD, and in our study, we verified their pharmacological efficacy by demonstrating their anti-proliferation, pro-apoptotic, hypoxia-modulating, and pro-lipid droplet synthesis effects on PASMCs. This suggests that neohesperidin and naringin could be potential chemical components of ZXGD contributing to its effectiveness against PH.

**Fig. 9:**
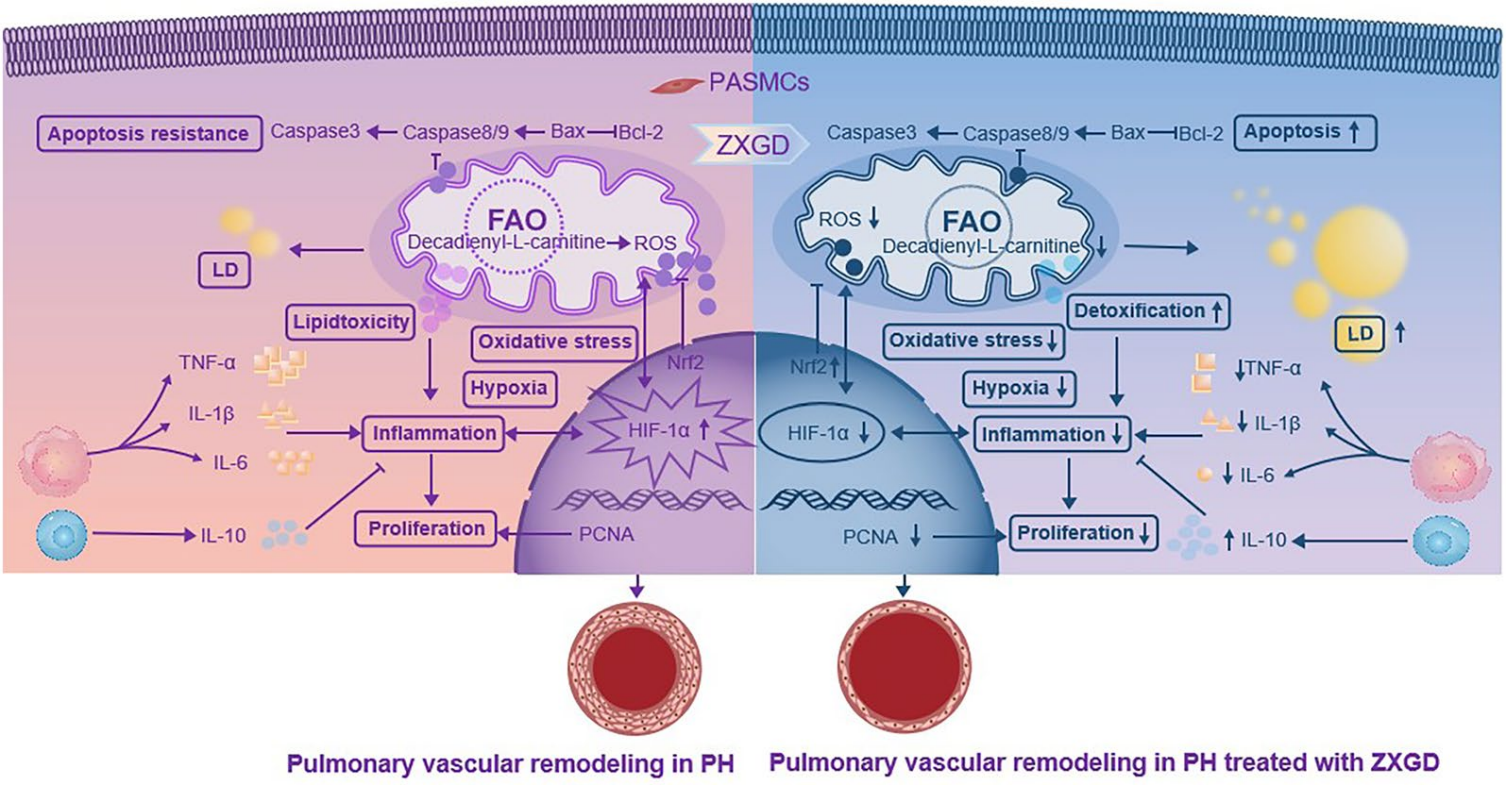
The pharmacological mechanism of ZXGD against pulmonary hypertension

Furthermore, we determined ZXGD exerts multiple pharmacological effects to alleviate pulmonary vascular remodeling, thus to ameliorate PH (Fig. 9). Apoptosis resistance and uncontrolled proliferation are typical traits of pulmonary vascular remodeling in patients with PH [49]. Excitingly, we confirmed ZXGD can ameliorate pulmonary vascular remodeling by reversing apoptosis resistance and excessive proliferation of PASMCs, thus maintaining pulmonary vascular homeostasis. This effect is conferred through the modulation of caspase 3, caspase 8, caspase 9, Bax, Bcl 2, and PCNA in vivo and in vitro. Moreover, we found HIF-1α was upregulated in PH rats induced with MCT (not hypoxia), indicating low oxygen availability, which leads to mitochondrial dysfunction, augmented ROS production, and Nrf2 downregulation, suggested the occurrence of oxidative stress [50–52]. Additionally, high levels of pro-inflammatory cytokines IL-6, IL-1β, and TNF-α, and low concentration of anti-inflammatory factor IL-10 confirm the presence of pulmonary vascular inflammation in PH rats. IL-6 is an independent predictor of survival in patients with PH, IL-1β can stimulate IL-6 production and promote the muscularization of distal small arteries [53], TNF-α can increase pulmonary artery pressure and pulmonary vascular remodeling [54], while IL-10 can protect against PASMC proliferation and prevent progression of PH [55]. Importantly, ZXGD could decrease these adverse factors such as HIF-1α, ROS, IL-6, IL-1β and TNF-α, while increase beneficial factors such as Nrf2 and IL-10, to relieve hypoxia, oxidative stress and inflammation to ameliorate pulmonary vascular remodeling in PH rats.

Excessive decadienyl-l-carnitine can cause mitochondrial uncoupling, increased ROS production, pro-inflammatory response, PASMC proliferation, and lipotoxic cardiomyopathy [24, 56, 57]. LDL-C, another harmful lipid, can accelerate disease progression by exacerbating inflammation and directly affecting pulmonary vasculature [34]. We found overexpression of PPARγ and phospholipase A2 (PLA2), and accumulation of decadienyl-l-carnitine and LDL-C in this study, indicating an increase in cell lipid toxicity [43, 58]. Lipid droplets (LDs) and mitochondria are critical organelles of lipid metabolism homeostasis. The formation of LDs is an important mechanism for preventing accumulation of harmful acylcarnitines and protecting against lipotoxic dysfunction of mitochondria [59]. In our study, ZXGD significantly increased counts of LDs and expression of its marker protein PLIN2, inhibited overexpression of PPARγ and phospholipase A2 (PLA2), and reduced level of decadienyl-l-carnitine. This promotes the formation of first-line intracellular defense mechanism that safely stores decadienyl-l-carnitine in LDs and protects against mitochondrial damage and cell toxicity. The modulatory mechanism of ZXGD on cholesterol metabolism may involve upregulating HDL-C, downregulating TC and LDL-C in serum, promoting storage of cholesterol esters in LDs, and restoring counts of LDs in PASMCs, thus maintaining cholesterol metabolic homeostasis and enhancing cellular detoxification [60], thereby inhibiting lipid metabolism driving factors for pulmonary vascular remodeling in PH rats.

Importantly, we discovered that HIF-1α, as the core target of the therapeutic targets network of ZXGD against PH, can directly modulate downstream crucial targets PCNA, caspase 3, IL-6, IL-10, LDL-C, decadienyl-l-carnitine, and PLIN2 to alleviate pulmonary vascular remodeling.

## Conclusion

This study unveils the pharmacological impact and molecular mechanism of ZXGD in combating PH through the regulation of pulmonary vascular remodeling in rats. Significantly, neohesperidin and naringin have been identified and confirmed as key functional compounds responsible for ZXGD's intervention in PH. Our research provides empirical evidence on therapeutic potential, functional compounds, and pharmacological mechanisms of ZXGD, laying a robust groundwork for the future development of novel drugs derived from ZXGD for the treatment of PH.

## Supplementary Information

Supplementary Material 1.

## Data Availability

Data used to support the findings of this study are available from the corresponding author upon reasonable request.

## Abbreviations

ZXGD: Zhishi Xiebai Guizhi Decoction
PH: Pulmonary hypertension
PASMCs: Pulmonary artery smooth muscle cells
MCT: Monocrotaline
PDGF-BB: Platelet-derived growth factor-BB
PAT: Pulmonary arterial acceleration time
ET: Ejection time
α-SMA: α Smooth muscle actin
PLA2: Phospholipase A2
TC: Total cholesterol
LDL-C: Low-density lipoprotein cholesterol
HDL-C: High-density lipoprotein cholesterol
ROS: Reactive oxygen species
PLIN2: Perilipin 2
LDs: Lipid droplets
BPC: The base peak chromatogram
