# Supplementary material for: Zhishi Xiebai Guizhi Decoction modulates hypoxia and lipid toxicity to alleviate pulmonary vascular remodeling of pulmonary hypertension in rats
Source: Chin Med. 2024 Dec 19;19:173. doi: 10.1186/s13020-024-01039-0 (PMC11657759; doi:10.1186/s13020-024-01039-0)
Supplement: Supplementary file 1 — Supplementary Material 1. [file 13020_2024_1039_MOESM1_ESM.pdf]

Supplementary Data

Tables S1-S2 and Figures S1-S8

Zhishi Xiebai Guizhi Decoction Modulates Hypoxia and Lipid toxicity to Alleviate Pulmonary Vascular Remodeling of Pulmonary Hypertension in Rats

Min Fu [a, #], Yuan Li [a, #], Jingjing Liu [c, d, #], Junjie Liu [a, #], Jiaoxia Wei [a], Yuxin Qiao [a], Hanxin Zhong [a], Dongyang Han [a], Haitao Lu [c, d, *], Li Yao [a, b, *]

[a] Department of Medicinal Chemistry and Natural Medicine Chemistry, Department of Pharmacognosy, College of Pharmacy, Harbin Medical University, Harbin 150081, China

[b] State-Province Key Laboratory of Biomedicine-Pharmaceutics of China, Harbin Medical University, Harbin 150081, China

[c] School of Chinese Medicine, State Key Laboratory of Environmental and Biological Analysis, Hong Kong Chinese Medicine Phenome Research Center, Hong Kong Baptist University, Hong Kong 999077, China

[d] Key Laboratory of Systems Biomedicine (Ministry of Education), Shanghai Center for Systems Biomedicine, Shanghai Jiao Tong University, Shanghai 200240, China

# These authors contributed equally to this work

***Corresponding authors**

Li Yao, Harbin Medical University, 157 Baojian Road, Nangang District, Harbin, 150081, China; E-mail: yaol@hrbmu.edu.cn;

Haitao Lu, Hong Kong Baptist University, 7 Baptist University Road, Kowloon Tong, Hong Kong 999077, China; Email: haitaolyu@hkbu.edu.hk

Table S1: Key parameters of QTOF-MS

Parameters	Values
Sheath gas temperature	350°C
Sheath gas flow rate	12L/min
Dry gas temperature	300°C
Dry gas flow rate	10L/min
Capillary voltage in positive mode	4000V
Capillary voltage in negative mode	3500V
Nozzle voltage	2000V
Nebulizer gas pressure	40psi

Table S2: Information for 18 precision identified components of ZXGD

CP	Name	RT	m/z	CE
1	Neohesperidin	8.4	611.3002	25
2	Naringin	7.98	581.2233	10
3	Adenosine	1.59	268.1049	10
4	Naringenin	7.98	273.0762	25
5	Adenine	1.58	136.0616	25
6	Limonin	14.24	471.2014	25
7	Isosakuranetin	10.18	287.0913	25
8	Xanthotoxol	9.15	203.0341	25

9	Eriocitrin	7.17	597.1803	25
10	Hesperitin	8.30	303.0859	25
11	Poncirin	10.22	595.2012	10
12	Scopoletin	6.97	193.0504	25
13	Syringic acid	5.48	197.0456	25
14	Esculetin	4.56	177	25
15	Neoeriocitrin	7.27	595.1658	40
16	Magnoloside A	6.42	623.1976	25
17	Magnoloside B	6.02	785.2499	40
18	Lonicerin	10.15	593.1865	40

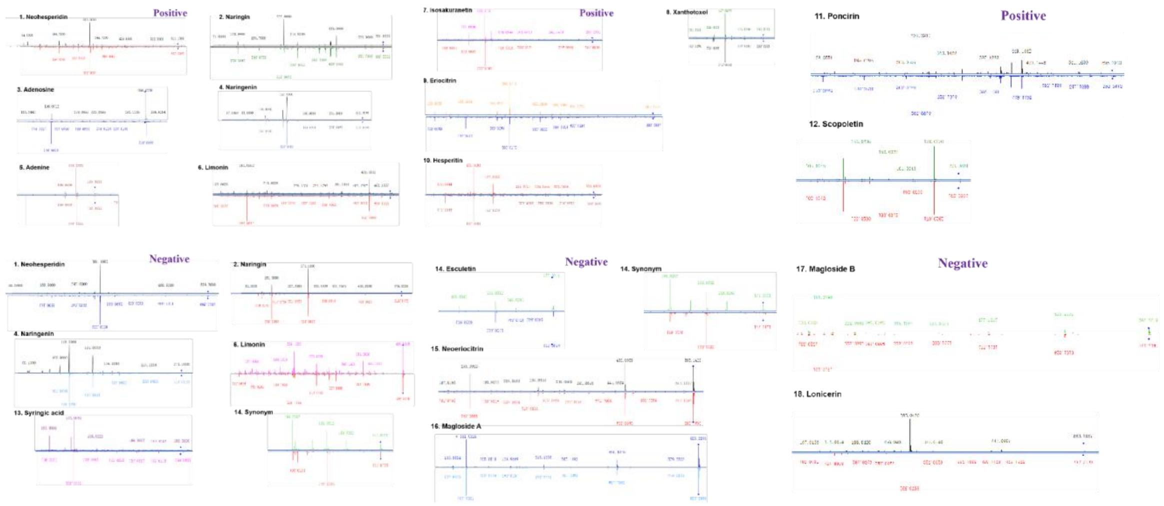

7. Isosakuranetin (CAS:480-43-3 , mw:286.28)

Reference compound：pos , CE: 25V

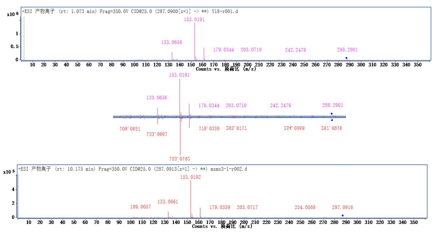

Sample：pos , CE: 25V

11. Poncirin, (CAS:14941-08-3 , mw:594.56)

Reference compound：pos , CE: 10V

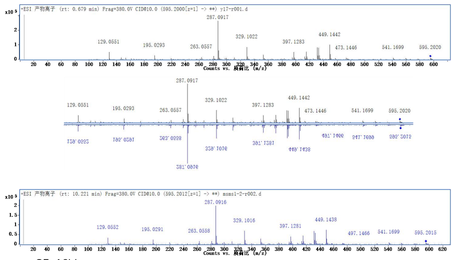

Sample：pos , CE: 10V

Figure S1 MS spectra of 18 identified compounds of ZXGD

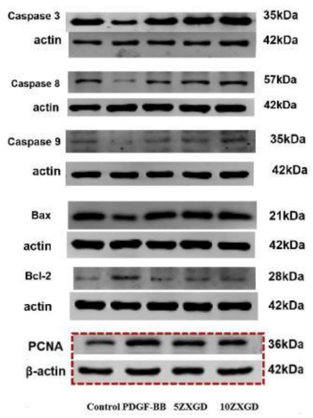

Figure S2 The original bands of proliferation and apoptosis protein expression in PASMCs in western-blot experiment.

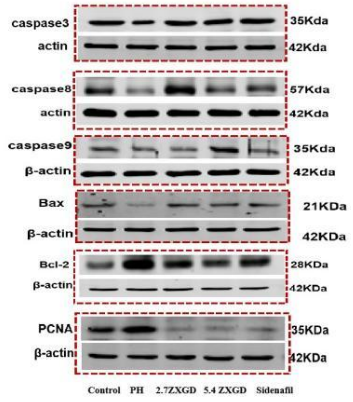

Figure S3 The original bands of proliferation and apoptosis protein expression in lung tissue of rats in western-blot experiment.

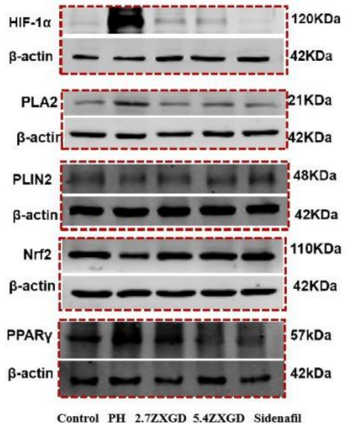

Figure S4 The original bands of HIF-1α, PLA2, PLIN2, Nrf2, PPARγ expression in lung tissue of rats in western-blot experiment.

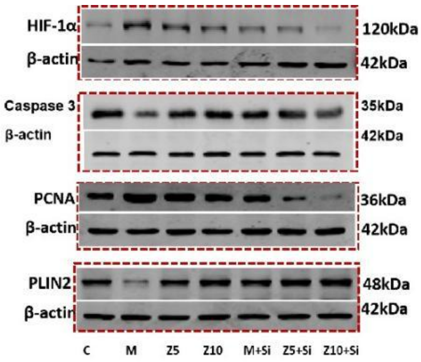

Figure S5 The original bands of HIF-1α, Caspae3, PCNA, PLIN2 expression in PASMCs transfected with HIF-1α siRNA in western-blot experiment.

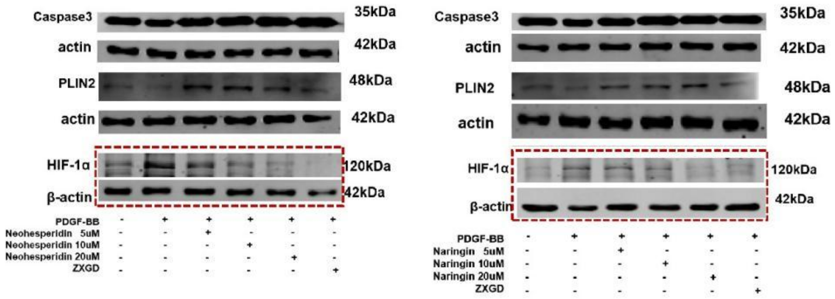

Figure S6 The original bands of Caspase3, PLIN2 and HIF-1α expression in PASMCs exposed to Neohesperidin and Naringin in western-blot experiment.

Molecular docking verification

HIF-1α was selected as a target for molecular docking with 18 chemical compounds. The chemical structures of 18 compounds were downloaded from PubChem database. They were imported into Open Babel 3.1.1 to convert format to mol2, and then imported into AutoDockTools 1.5.6 for processing, and files were saved in pdbqt format. The 3D crystal structure of target protein was downloaded from PDB protein

database (https://www.rcsb.org/) with PDBIDs 5I9V. The water molecule and organic matter in target protein were removed by Pymol, and then target protein was imported into Auto DockTools1.5.6 for hydrogenation, charge distribution, and atomic type addition and saved in pdbqt format. Molecular docking was performed using AutoDockVina and docking results were plotted using Pymol.

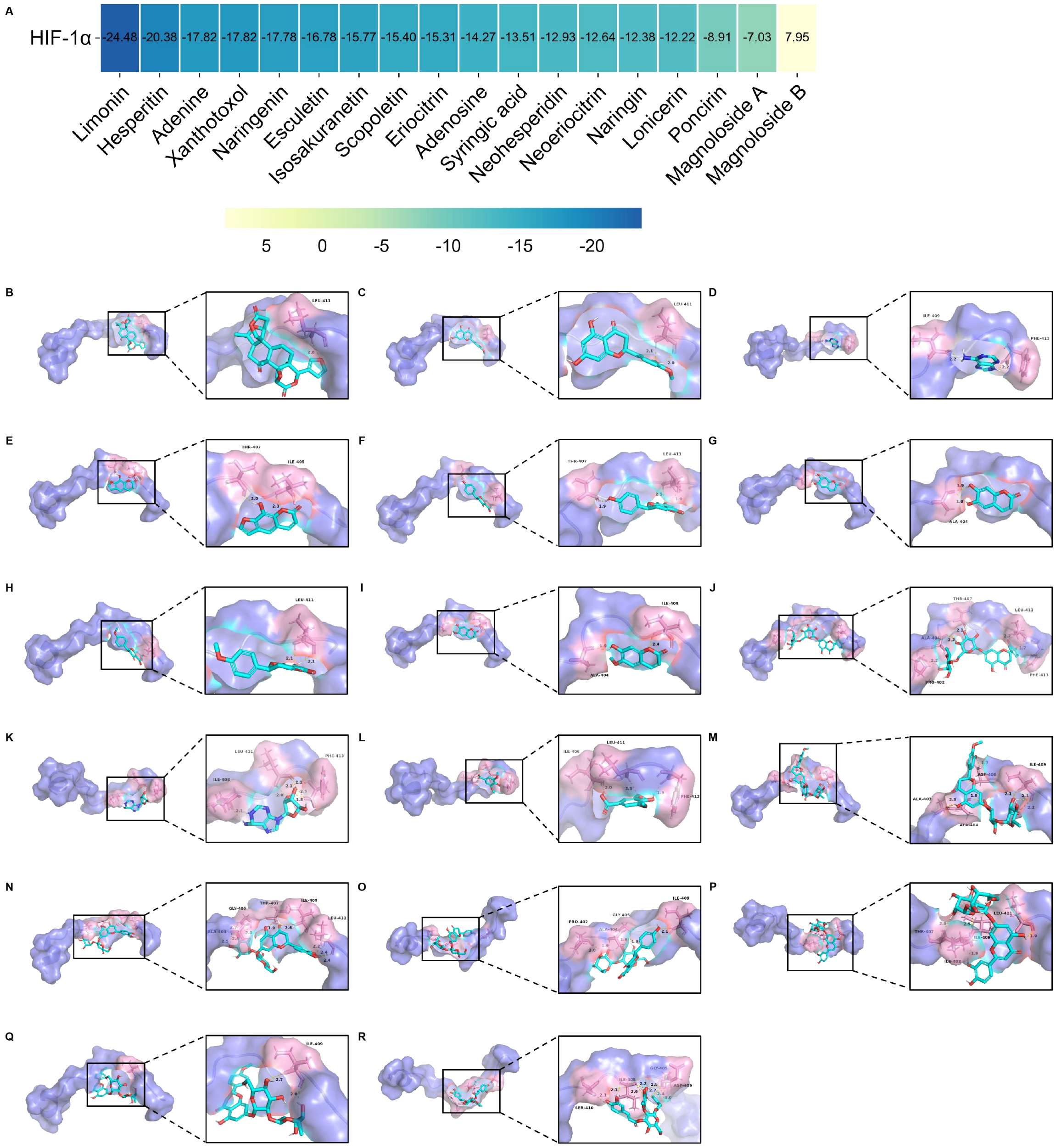

Figure S7. Molecular docking results of 18 chemical compounds of ZXGD.

(A)Binding energy of compounds and HIF-1α. (B)HIF-1α-Limonin. (C) HIF-1α-Hesperitin. (D)HIF-1α-Adenine. (E)HIF-1α-Xanthotoxol. (F) HIF-1α-Naringenin. (G)HIF-1α-Esculetin. (H)HIF-1α-Isosakuranetin. (I) HIF-1α-Scopoletin. (J)HIF-1α-Eriocitrin. (K)HIF-1α-Adenosine. (L) HIF-1α-Syringic acid. (M)HIF-1α-Neohesperidin. (N)HIF-1α-Neoeriocitrin. (O)HIF-1α-Naringin. (P)HIF-1α-Lonicerin. (Q)HIF-1α-Poncirin. (R) HIF-1α-Magnoloside A.

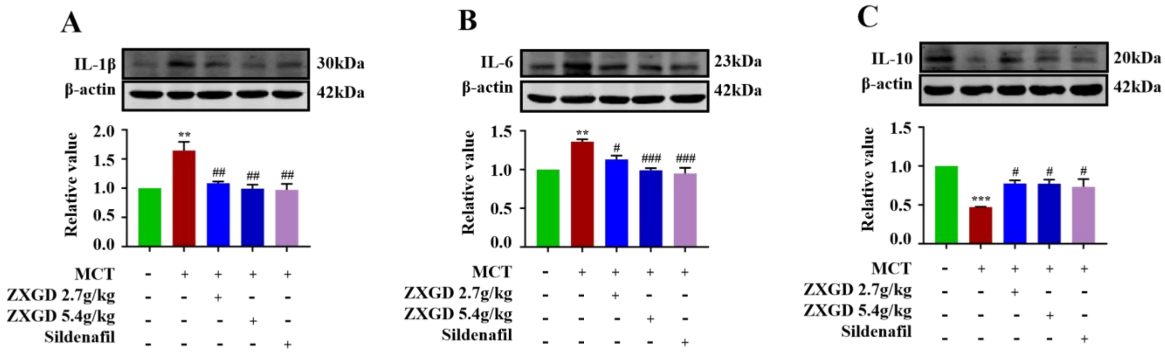

Figure S8. ZXGD reversed inflammatory factors IL-1β , IL-6 and IL-10 in lung tissue of rats with PH examined by Western blot. **p< 0.01, ***p< 0.001 vs control, #p< 0.05, ##p< 0.01, ###p< 0.001 vs MCT group.
